# The Complex Interplay of Insulin Resistance and Metabolic Inflammation in Transition Dairy Cows

**DOI:** 10.3390/ani14060832

**Published:** 2024-03-08

**Authors:** Kaixi Qiao, Renjiao Jiang, Genaro Andres Contreras, Lei Xie, Osvaldo Bogado Pascottini, Geert Opsomer, Qiang Dong

**Affiliations:** 1 College of Veterinary Medicine, Northwest A&F University, Yangling, Xianyang 712100, China; qkx06100610@126.com (K.Q.); jrj15318559019@163.com (R.J.); 2 Department of Large Animal Clinical Sciences, Michigan State University, East Lansing, MI 48824, USA; contre28@msu.edu; 3 Department of Internal Medicine, Reproduction and Population Medicine, Faculty of Veterinary Medicine, Ghent University, 9820 Merelbeke, Belgium; lei.xie@ugent.be (L.X.); osvaldo.bogado@ugent.be (O.B.P.); geert.opsomer@ugent.be (G.O.)

**Keywords:** insulin resistance, metabolic inflammation, transition dairy cows, lipid mobilization, adipokine

## Abstract

**Simple Summary:**

This review critically examined the literature on the interaction between insulin resistance (IR) and metabolic inflammation in transition dairy cows. Our review emphasizes how IR and metabolic inflammation mutually influence each other, leading to heightened lipolysis, immune activation, and tissue inflammatory pathways. These processes contribute to a harmful cycle where inflammatory mediators exacerbate IR and metabolic inflammation. While transient IR and metabolic inflammation are natural adaptations in transitioning cows, this review highlights the increased disease risk in over-conditioned cows. Understanding these interactions is crucial for managing metabolic disorders in dairy herds and promoting animal health, welfare, and productivity.

**Abstract:**

During the transition period, dairy cows exhibit heightened energy requirements to sustain fetal growth and lactogenesis. The mammary gland and the growing fetus increase their demand for glucose, leading to the mobilization of lipids to support the function of tissues that can use fatty acids as energy substrates. These physiological adaptations lead to negative energy balance, metabolic inflammation, and transient insulin resistance (IR), processes that are part of the normal homeorhetic adaptations related to parturition and subsequent lactation. Insulin resistance is characterized by a reduced biological response of insulin-sensitive tissues to normal physiological concentrations of insulin. Metabolic inflammation is characterized by a chronic, low-level inflammatory state that is strongly associated with metabolic disorders. The relationship between IR and metabolic inflammation in transitioning cows is intricate and mutually influential. On one hand, IR may play a role in the initiation of metabolic inflammation by promoting lipolysis in adipose tissue and increasing the release of free fatty acids. Metabolic inflammation, conversely, triggers inflammatory signaling pathways by pro-inflammatory cytokines, thereby leading to impaired insulin signaling. The interaction of these factors results in a harmful cycle in which IR and metabolic inflammation mutually reinforce each other. This article offers a comprehensive review of recent advancements in the research on IR, metabolic inflammation, and their intricate interrelationship. The text delves into multiple facets of physiological regulation, pathogenesis, and their consequent impacts.

## 1. Introduction

The peripartum period is a distinct physiological phase for dairy cows, during which they are susceptible to typical peripartum diseases such as ketosis and fatty liver. A study conducted by Santos et al., which evaluated 5719 lactations, concluded that 44% of cows experienced peripartum diseases within 60 days postpartum [1]. Extensive research conducted by diverse scientists has led to a focus on metabolism and the complex contribution of adipose tissue (AT) within the cow’s system. Current understanding suggests that periparturient diseases are primarily triggered by the negative energy balance (NEB) experienced during these critical periods. Metabolic inflammation and insulin resistance (IR) play a fundamental role in driving the pathogenesis of these disorders [2,3,4,5]. This review aims to provide a comprehensive evaluation of the literature on metabolic inflammation, IR, and the intricate relationship between the two in transition dairy cows.

## 2. Regulation of Metabolic Function during the Peripartum Period

### 2.1. The Regulatory Role of Insulin in Glucose and Lipid Metabolism and the Development of Insulin Resistance

**Figure 1:**
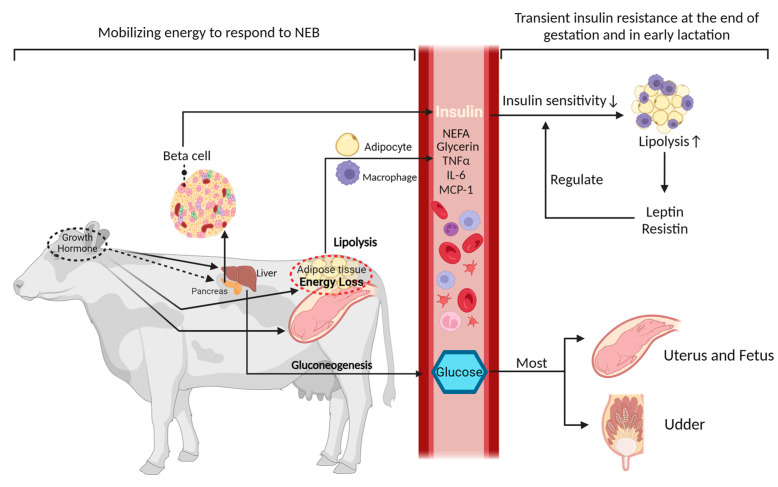
Metabolic dynamics and hormonal interplay during the peripartum period in dairy cows. During the peripartum period, growth hormone increases as lactation begins, which indirectly affects insulin levels and sensitivity, and also triggers lipolysis in adipose tissue to release energy reserves [9]. This response plays a crucial role in fetal development, milk secretion, and the initiation of lactation. During the transition from late pregnancy to early postpartum, dairy cows exhibit a short period of IR. This is predominantly demonstrated by the stimulation of the hepatic gluconeogenesis process, which directs most of the glucose toward sustaining the growth of the fetus and milk production [8]. Concurrently, alterations in adipokines, such as leptin and resistin, stimulate increased lipolysis in adipose tissue, leading to an elevation in the release of free fatty acids. These adipokines are essential in modulating insulin sensitivity, either by enhancing or diminishing it [10,11].

Insulin, secreted by the pancreatic beta cells (Figure 1), is the hormone responsible for directly lowering blood glucose levels and stimulating energy storage [6]. It facilitates the synthesis and storage of glycogen, lipids, and proteins [6]. Insulin inhibits glycogenolysis in the liver and skeletal muscle, and it also suppresses lipolysis in AT, resulting in reduced levels of circulating fatty acids [7]. This dual mechanism facilitates the uptake and utilization of glucose, thereby effectively regulating blood glucose levels within the normal range [7,8].

Throughout the transition from the pregnant, non-lactating state to subsequent lactation, the dynamics of insulin play a vital role in regulating glucose metabolism, energy balance, and milk production in the pre- and postpartum periods. Cows exhibit approximately four times higher basal insulin levels and insulin response to a glucose tolerance test (GTT) before compared to after calving, as evidenced by peak insulin concentrations and insulin increment indices [12,13,14]. Studies illustrated an increased demand for energy and nutrients to support the availability of nutrients, particularly glucose and fatty acids, for the developing fetus. This increased demand leads to a decrease in insulin sensitivity in peripheral tissues, leading to IR. Meanwhile, IR prompts increased lipolysis in AT, leading to an elevated release of non-esterified fatty acids (NEFA) into the bloodstream [15]. NEFAs serve as a significant energy source for the cow and contribute to the development of hepatic ketogenesis. Concurrently, insulin is well known as the primary hormone that suppresses hepatic gluconeogenesis and facilitates glucose uptake by peripheral tissues through the promotion of the translocation of glucose transporters [16,17]. During the regulation of gluconeogenesis, insulin exerts regulatory effects on the secretion and action of other hormones. For example, heightened blood levels of growth hormone (GH) can trigger a reduction in insulin sensitivity, leading to the development of IR [18]. This is illustrated by GH’s stimulation of gluconeogenesis in the liver by increasing the utilization of amino acids and glycerol as substrates. The latter enhances glucose production and leads to higher glucose levels in the peripheral circulation (Figure 1) [9,19]. By this higher peripheral glucose levels, GH in fact contributes to a situation that resembles physiologic insulin resistance, which refers to a very similar situation. Due to the very high and insulin-independent uptake of glucose by the udder, the typical clinical features of hyperglycemia as seen in human medicine are less frequently seen in high-yielding dairy cows. Insulin counteracts the effects of GH by promoting glucose uptake and suppressing lipolysis. Meanwhile, insulin primarily reduces gluconeogenesis by suppressing the expression of key gluconeogenic enzymes, such as pyruvate carboxylase (PC) and phosphoenolpyruvate carboxykinase (PEPCK) [12]. In the context of NEFA-induced lipidosis of the liver and ketosis, the uncoupling of the GH-insulin-like growth factor 1 (IGF-1) axis and its potential relationship with inflammation and low IGF-1 levels is a complex topic [20]. This uncoupling, characterized by elevated GH, reduced expression of hepatic growth hormone receptor (GHR), and diminished IGF-1 levels, affects liver function and metabolic health, potentially leading to conditions such as liver lipidosis and ketosis [20]. Notably, low IGF-1 levels have been associated with various metabolic disturbances, including IR, impaired glucose tolerance, and dyslipidemia [21,22]. Furthermore, IGF-1 modulates the differentiation and proliferation of myeloid lineage cells and affects the responsiveness of mature immune cells to antigens [23]. Cows with high IGF-1 levels had better nutrient availability and could more effectively use their adaptive immune system to resist infections, which is compromised in high-yielding dairy cows due to the uncoupling of the GH-IGF1-axis [23].

**Figure 2:**
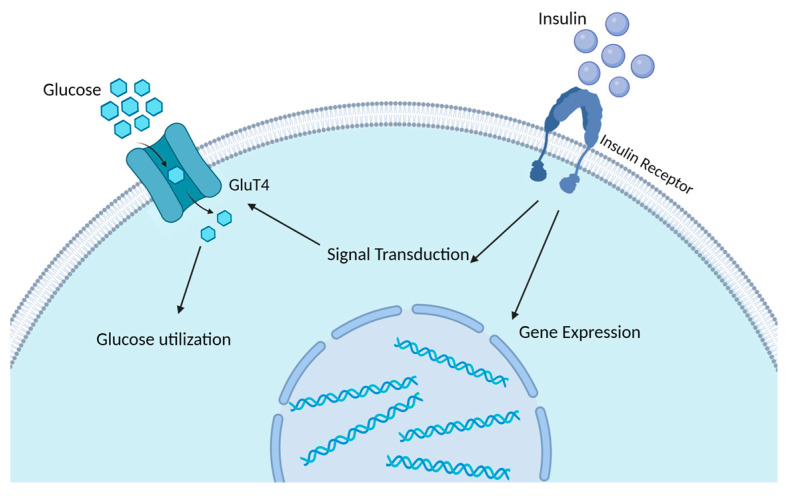
Insulin signaling initiation and glucose uptake machinery in adipocytes. The initiation of insulin signaling occurs when insulin binds to the insulin receptor substrate, subsequently triggering downstream signals that enhance the gene and protein synthesis of glucose transporter-4 (Glut4). The Glut4 protein facilitates the transportation of glucose across the cell membrane, thereby enabling its entry into the cell for metabolic utilization. The initiation and culmination of insulin signaling pathways crucial for facilitating glucose uptake are marked by the insulin receptors and glucose transporters, respectively [16,17].

Elevated insulin levels suppress the activity of hormone-sensitive lipase (HSL), thereby inhibiting the release of fatty acids from adipocytes and consequently reducing the circulating levels of free fatty acids in the bloodstream. During the process of lipogenesis, insulin facilitates the transportation of glucose into adipocytes by promoting the translocation of glucose transporters (Figure 2) [16,17]. Insulin also stimulates the activity of enzymes responsible for the transformation of glucose into fatty acids, including acetyl-CoA carboxylase and fatty acid synthase [24]. Additionally, upon entering the adipocyte, glucose undergoes glycolysis to generate glycerol, which serves as the backbone for triglyceride synthesis [25]. Reduced circulating insulin in combination with increased concentrations of GH, however, is a significant trigger of lipolysis, resulting in the breakdown of triglycerides into free fatty acids (NEFA) and glycerol (Figure 1). NEFA is enzymatically cleaved from triglyceride molecules within adipocytes through the activity of HSL (Figure 1) [26]. Hence, insulin plays a central role in regulating both the lipolysis and lipogenic pathways and is, therefore, crucial in the homeorhetic adaptations in transition dairy cows.

During the late stages of pregnancy in cows, the increased fetal growth, along with an irregular insulin-independent uptake of glucose by the pregnant uterus (Figure 1) [8], lead to an approximate glucose requirement of 0.10 mol/kg fetus/d in the pregnant uterus [27]. During the latest stages of pregnancy, cows also undergo significant hormonal changes, like increased levels of cortisol, which affect blood glucose concentration in a way opposite to that of insulin, impairing glucose disappearance [28,29]. After parturition, a rapid increase in milk production is observed in dairy cows without a corresponding increase in dry matter intake (DMI), leading to an NEB. This situation is particularly pronounced in high-yielding cows, which are unable to sufficiently increase their DMI to meet the energy demands of milk production in the immediate postpartum period [30]. This NEB results in the mobilization of body reserves, particularly fat [31]. Under the situation of the high yield, the reduced-DMI-caused NEB is the cause. The NEB state can precipitate various health issues, as the cow’s body mobilizes fat reserves, leading to metabolic disorders. Health problems, the result, in turn, can worsen NEB by reducing feed intake or nutrient absorption, or by increasing energy requirements for fighting against infections or healing [32,33]. The process of lipid mobilization also leads, besides the increase in circulating NEFAs, to the elevated secretion of adipokines such as leptin and resistin (Figure 1) [10,11,15]. The latter further contributes to impaired insulin sensitivity, resulting in IR and the initiation of inflammatory responses [15]. The inefficient hepatic processing of these NEFAs leads to lipidosis, commonly known as fatty liver, and the production of ketone bodies, indicative of ketosis [31]. In the study of Arshad and Santos, as the concentration of hepatic triacylglycerol increased, there was an increase in milk yield and energy-corrected milk (ECM), but this came at the expense of body reserves, as indicated by exacerbated losses of body weight and a more negative body energy change [34]. This situation was associated with reduced intakes of dry matter (DM) and net energy for lactation (NEL), alongside increases in blood levels of NEFA and BHB, and decreases in glucose and total calcium [32,34]. During the initiation of lactation, insulin only regulates around 8% of the blood glucose uptake, since most of the glucose at that time is diverted to the mammary gland in an insulin-independent manner [35]. Despite reduced DMI and low glucose and insulin concentrations, there is a persistent supply of glucose to the udder for milk synthesis, requiring approximately 3.2 kg of glucose for the production of 45 kg of milk [36]. The mammary gland’s heightened glucose utilization leads to reduced blood glucose, triggering lipolysis to meet the increased energy demands, reflecting the disrupted insulin levels and sensitivity in the peripheral tissues [35]. This homeorhetic regulatory mechanism collectively defines the state of IR, which is characterized by a shift of glucose allocation toward the gravid uterus and udder, while the cow’s body upregulates glucose production through gluconeogenesis to maintain blood glucose homeostasis (Figure 1). The transformation of the ‘excess’ of glucose into lactose in the lactating cows’ udders results in a milder disruption of the glucose metabolism in comparison to the lipid metabolism during IR [37]. This statement elucidates the rationale behind the increased likelihood of noticeable IR in over-conditioned dairy cows, whose DMI is lower [38,39,40]. While the heightened lipid flux provides extra energy during periods of NEB, an excessive transformation of NEFAs into ketone bodies surpasses the metabolic capacity of the cow to efficiently utilize ketone bodies as an energy source, increasing the risk of ketosis [41]. The study by Zhang et al. on ketotic cows with abnormal and normal glucose tolerance revealed a significant correlation between the occurrence of ketosis in cows with abnormal glucose tolerance and dysregulated glucose utilization due to IR. Abnormal liver function and heightened oxidative stress were furthermore identified as contributing factors to the occurrence of IR [42].

In summary, the transition period in dairy cows involves intricate insulin dynamics, playing a crucial role in regulating glucose metabolism, energy balance, and milk production, while factors such as lipolysis, lipogenesis, and multiple other hormonal changes further contribute to IR, influencing metabolic outcomes during the transition from non-lactating to lactating states.

### 2.2. Metabolic Inflammation in Transition Dairy Cows

During the periparturient phase in dairy cows, shifts in energy balance, hormonal changes, and nutrient metabolism are associated with the development of subacute inflammation, also referred to as metabolic inflammation [43]. Metabolic inflammation refers to a low-grade or mild inflammatory response induced by metabolic stress [44]. Chronic systemic inflammation exacerbates the susceptibility to infectious diseases, including mastitis and metritis [45]. Not all cows undergo chronic inflammation during the transition period, but the risk is higher, particularly in over-conditioned cows [5,46]. This chronic inflammation interferes with insulin action, elevating the risk of metabolic disorders and activating multiple types of immune cells, thereby causing subtle increases in inflammatory mediators, leading to persistent alterations in tissue functionality and a state of low-grade inflammation [43]. The primary mechanisms that contribute to metabolic inflammation involve elevated levels of NEFAs and pro-inflammatory adipokines resulting from lipolysis and oxidative stress [47,48,49,50].

**Figure 3:**
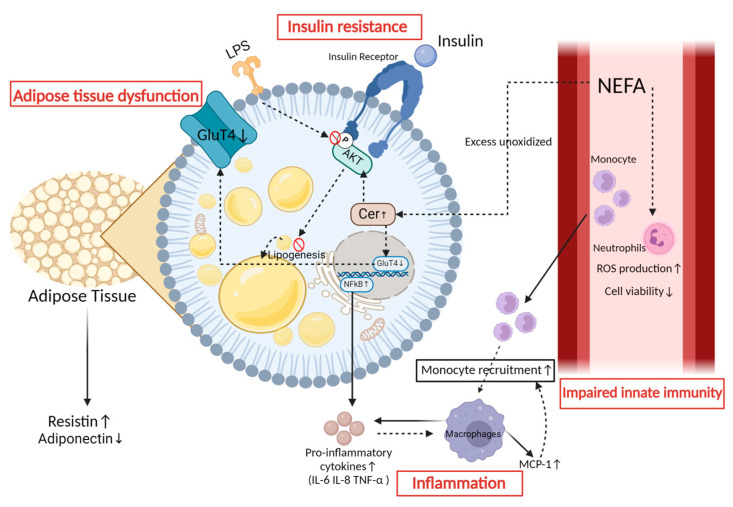
Impacts of lipid mobilization in adipose tissue. Lipid mobilization in the adipose tissue leads to four specific consequences. (1) Inflammation: In adipocytes, the regulation of NF-κB results in the production of pro-inflammatory factors [74,75]. These factors contribute to the polarization of macrophages toward the M1 phenotype and the secretion of monocyte chemotactic protein 1, which facilitates the recruitment of monocytes and their subsequent differentiation into macrophages [76]. (2) Impaired innate immunity: NEFA can stimulate an increase in ROS production and decrease cell viability in neutrophils in vitro [53,54]. (3) Insulin resistance: Insulin resistance can be attributed to the persistent elevation of NEFA, leading to an increase in ceramide concentration. The elevated levels of this factor impede the insulin-stimulated uptake of glucose by reducing the activation of protein kinase B (Akt) in primary bovine adipocytes. Additionally, phosphorylation of Akt is hindered during LPS infection, leading to the suppression of Akt activation [69,77]. (4) Adipose tissue dysfunction: Adipocytes secrete resistin in response to pro-inflammatory factors, while also decreasing the synthesis of adiponectin, thus impacting the overall functionality of adipose tissue [11,78].

Lipolysis triggers an increase in NEFAs, which has the potential to engage with intracellular signaling pathways in various cell types, thereby inducing inflammation [51]. The typical NEFA, for instance, overstimulates the Toll-like receptor (TLR)—the nuclear factor-κB (NF-κB) inflammatory signaling pathway—in bovine neutrophils in vitro, causing increased expression and phosphorylation of TLR2, TLR4, and NF-κB p65, thereby promoting the expression of the pro-inflammatory cytokines interleukin-1β (IL-1β), IL-6, and tumor necrosis factor-alpha (TNF-α) [52]. NEFAs have also been shown to stimulate in vitro neutrophils, leading to an increase in ROS generation and a decrease in cell viability (Figure 3) [53,54]. In the study by Vanacker et al., the lipid infusion model was designed to mimic the metabolic conditions surrounding parturition, revealing that elevated NEFA levels have a direct impact on immune function [55]. This was evidenced by decreased lymphoproliferation and reduced secretion of interferon-γ in peripheral blood mononuclear cells, along with a diminished oxidative burst in polymorphonuclear neutrophils [55]. These effects occur independently of the hormonal and metabolic shifts typically seen during parturition, highlighting the pivotal role of NEFAs in triggering and maintaining inflammation, regardless of the stress associated with parturition. Furthermore, NEFA supplementation stimulates in vitro endothelial cells, leading to increased levels of IL-6 and IL-8, as well as increased generation of reactive oxygen species (ROS) and changes in the phospholipid fatty acid profile [56]. This process induces the production of metabolites derived from linoleic acid, including 9- and 13-hydroxyoctadecadienoic acid, which are recognized for their pro-inflammatory characteristics [56]. Moreover, elevated concentrations of palmitic acid, a principal saturated fatty acid among peripheral NEFA, activate the NF-κB signaling pathway in bovine endometrial cells, increasing the expression of pro-inflammatory cytokines IL-8, IL-6, and TNF-α, thereby amplifying the inflammatory response [57]. Meanwhile, the metabolic inflammation in transition dairy cows is also stimulated by the secretion of adipokines, which is enhanced by AT mobilization. In AT, there is an increased release of pro-inflammatory adipokines, such as monocyte chemotactic protein 1 (MCP-1), accompanied by a simultaneous decrease in the release of anti-inflammatory adipokines, such as adiponectin [58,59].

Moreover, the heightened metabolic demands and lipid oxidation in dairy cows lead to an elevated generation of ROS, which are the by-products of cellular metabolism [47]. This rise temporarily depletes the body’s antioxidant mechanisms, rendering it susceptible to oxidative stress, ultimately resulting in heightened metabolic inflammation [47,60,61]. Elevated levels of NEFAs in bovine hepatocytes lead to increased ROS generation, activating the c-Jun N-terminal kinase (JNK) pathway, which in turn, triggers the activation of p53 transcription and the suppression of Nrf2 transcription, ultimately depleting the mitochondrial membrane potential [62]. As a result, the release of apoptosis-inducing factor and cytochrome c into the cytoplasm facilitates liver cell apoptosis [62]. Furthermore, mitochondrial ROS activation induces the NOD-like receptor protein 3 (NLRP3) inflammatory response under NEFA-induced metabolic stress, mediating apoptosis in bovine mammary epithelial cells in vitro [63]. Xudong Sun and colleagues observed a decline in the levels of glutathione peroxidase, superoxide dismutase, and catalase in the mammary glands of ketotic cows, accompanied by a reduction in NF-κB signaling and NLRP3 inflammasome activation [64].

In addition to nutritional interventions, addressing the genetic factors underlying energy balance traits is crucial for sustainable dairy cow management. Studies have focused on the genetic correlation between milk yield and energy balance for decades [65] and observed a moderate negative genetic correlation between milk yield and energy balance in early lactation [66]. This suggests that cows selected for higher milk production may experience increased NEB unless the energy balance is directly or indirectly considered in breeding programs [30]. Therefore, NEB in modern high-genetic-merit dairy cows with higher milk yields is considered a man-made problem. Unlike in beef cattle where NEB may be considered natural, continuous selection for higher milk production in dairy cows has exacerbated NEB, leading to significant energy deficits. However, since the measurement of DMI is difficult and the corresponding data for genetic selection are not sufficiently available, the energy balance (indicator) itself is not typically considered a direct trait for genetic selection in dairy cattle breeding programs [31].

Furthermore, in cases of infectious diseases such as mastitis, the presence of endotoxins, a classical source of infectious inflammation, can intensify metabolic inflammation and weaken the response of the immune system, which is complex [67,68]. A previous study on in vitro AT from dairy cows has demonstrated that lipopolysaccharide (LPS) activates inflammatory lipolytic pathways and inhibits Akt phosphorylation, leading to decreased insulin sensitivity, and also triggers the MEK/ERK signaling pathway, resulting in significant transcriptional upregulation of IL-6 and IL-8 (Figure 3) [69]. Additionally, the significant activation of MEK/ERK induced by LPS may have an impact on the β-3 adrenergic receptors, resulting in the phosphorylation of HSL and consequently promoting lipolysis [69,70,71]. In human medicine, strong lipid mobilization leads to the downregulation of AT-resident regulatory T cells and the IL-4-producing eosinophils, which are associated with antimicrobial functions [72]. Horst et al. administered LPS intravenously to cows, either alone or in combination with lipids [73]. Their findings revealed that the infusion of LPS alone triggered a distinct metabolic response characterized by transient hyperinsulinemia, with insulin levels peaking significantly post-infusion before declining. Concurrently, lipid infusion, whether administered alone or in conjunction with LPS, led to elevated serum triglyceride levels. Notably, when LPS was combined with lipid infusion, the increase in triglycerides was more pronounced. This observation suggests that LPS may exacerbate the lipid-induced rise in serum triglycerides, possibly through mechanisms that impair triglyceride clearance or enhance its production. Despite these alterations in serum insulin and triglyceride levels, liver triglyceride content did not significantly differ across treatments. The latter implies that acute systemic inflammation induced by LPS, even in the presence of hyperlipidemia, does not necessarily exacerbate liver fat accumulation in the short term.

## 3. The Mechanism of Insulin Resistance in Peripartum Dairy Cows

The molecular mechanisms of IR finally lead to hindered glucose uptake by insulin-sensitive tissues, increased lipolysis, and changes in sphingolipid metabolism. Insulin responsiveness is defined as the impact of insulin on target tissues, measured by the level of glucose uptake [8]. In contrast, insulin sensitivity is defined as the concentration of insulin required to achieve half of the maximum response [8]. As described in Figure 2, under normal physiological conditions, insulin binds to the insulin receptor located on the cellular membrane of insulin-sensitive tissues, primarily including muscle, AT, and the liver [16,79]. This interaction gives rise to a series of events that involve insulin receptor substrate signaling, ultimately leading to an increase in cellular glucose uptake and the activation of the phosphatidylinositol 3-kinase (PI3K)/protein kinase B (Akt) signaling pathway [80]. This process can increase the expression of the gene that is responsible for encoding the glucose transporter-4 (Glut4), promoting the translocation and integration of these components into the cell membrane, thereby facilitating the transportation of sugar into the cells (Figure 2). The insulin receptor and sugar transporter, respectively, mark the initiation and end of the insulin signaling-mediated process that promotes glucose uptake (Figure 2) [16,17]. Previous research in high-yielding postpartum cows has shown a strong correlation between IR in AT, plasma insulin levels, and the extent of Akt phosphorylation in AT [12]. Additionally, in the AT of postpartum cows, decreased levels of insulin receptor mRNA and protein were observed in over-conditioned cows, suggesting a potential reduction in insulin responsiveness to glucose [81]. In the study by Angeli et al., it was demonstrated that dairy cows with higher body condition scores (BCS) exhibited hepatic IR, as evidenced by lower levels of AKT phosphorylation [82].

The long-term effects of elevated NEFA levels have adverse effects, not only on pancreatic insulin secretion but also on the phosphorylation of serine residues on insulin receptor substrate-1 (IRS-1), leading to a reduction in the tyrosine phosphorylation of IRS-1 [83,84,85]. This disruption impairs the insulin signaling cascade and hinders its common activation, ultimately resulting in compromised insulin-mediated glucose uptake by peripheral tissues [83,84,85]. Moreover, NEFAs can trigger the TLR4/NF-κB inflammatory signaling pathway in hepatocytes, thereby reducing insulin sensitivity via the TLR4/PI3K/AKT metabolic axis in calf hepatocytes in vitro [86].

Ceramide (Cer) is known to play a critical role in the metabolism of sphingolipids. C2:0-Cer has been observed to impede the insulin-induced uptake of 2-Deoxy-D-glucose by diminishing Akt activation in primary bovine adipocytes (Figure 3) [77]. Moreover, it has been shown that Cer inhibits the expression of GLUT4 (Figure 3) [87]. Therefore, Cer serves as a crucial sphingolipid biomarker for IR in dairy cows. Elevated levels of circulating fatty acylcarnitines (FAC) are associated with Cer in over-conditioned dairy cows experiencing IR. Rico and colleagues showed that an excess of unoxidized NEFAs contributes to the accumulation of FAC and Cer, which is inversely associated with insulin sensitivity (Figure 3) [88]. Moreover, the fluctuations in lipid composition, as demonstrated by C16:0- and C24:0-Cer, are associated with a negative correlation with postpartum systemic insulin sensitivity [88,89].

## 4. The Impact of Metabolic Inflammation on Dairy Cows’ Health during the Periparturient Period

The inflammatory response in dairy cows during the periparturient period is a complex and crucial process that involves a coordinated series of immune responses aimed at protecting the cow from potential infections associated with parturition and facilitating the transition into lactation. Numerous researchers have investigated the effects of the immune-inflammatory response on the metabolic function of dairy cows [37,90]. Immunological activation, marked by increased leukocyte quantity and functionality, is essential for cervical dilatation and uterine contractions during parturition, with leukocyte infiltration into the cervix before and after delivery playing a crucial role in coordinating matrix remodeling through the release of proteolytic enzymes [91,92]. Monocytes and eosinophils demonstrate a progesterone-regulated rise in number in the cervix before delivery, while the number of neutrophils increases after delivery [91,92]. Neutrophils, in particular, exhibit a significant dependence on glucose metabolism to meet their metabolic requisites [43,93]. Consequently, in cases of metabolic inflammation, the initiation of inflammatory signals is a contributing factor in the development of IR, leading to a reorganization of metabolic resources [43,93]. This redirection effectively directs nutrients, which are usually used for normal physiological functions and production, to sufficiently support the quantity and functionality of immune cells.

Clinical trials suggest that repeated, transient, and sustained subacute inflammatory responses have a significant impact on the metabolic function of dairy cows. Kushibiki et al. conducted an investigation into the effects of administering recombinant TNF-α (rbTNF) once daily to mid-lactating dairy cows [94]. This intervention resulted in a 34% reduction in feed intake, elevated plasma NEFA concentrations, and a decrease in the plasma insulin-like growth factor 1 concentration [94]. Bradford and colleagues administered doses of rb TNF-α at levels lower than can be expected by subacute inflammation in late-lactation cows, revealing a twofold increase in liver triglyceride levels and a concomitant upregulation of lipid synthesis enzyme transcript abundance [95]. These findings suggest a direct effect of rbTNF on hepatic lipid metabolism [95]. The research by Ohtsuka et al. revealed higher TNF-α activity in severe compared to mild fatty liver cows [96]. Given that TNF-α could potentially interrupt insulin-stimulated tyrosine phosphorylation in insulin transmembrane signaling [97], this finding indicates the importance of TNF-α in the pathogenesis of IR observed in cows with fatty liver [96].

Furthermore, an increase in the mRNA levels of TNF-α was observed in the AT of peripartum dairy cows [98,99]. In particular, cows with high postpartum BCS losses exhibited the highest levels of the mRNA abundance of genes encoding IL-6 and TNF-α in the AT at 21 and 42 days postpartum [99]. The signaling of TNF-α occurs via TNF receptors 1 and 2, leading to transcriptional alterations facilitated by the NF-κB and extracellular ERK signaling pathways [100]. In a study conducted by Martel et al., stable and low-level recombinant TNF-α was administered to the subcutaneous fat of late-lactating dairy cows for 7 consecutive days in a clinical trial [101]. The findings indicated that the levels of triiodothyronine and insulin-like growth factor 1 were reduced in the treatment group, indicating that the intervention by TNF-α significantly affects the metabolism [101].

## 5. The Relationship between Insulin Resistance and Metabolic Inflammation

Within the dynamic periparturient phase of dairy cows, the intricate dance of IR and metabolic inflammation, orchestrated by pivotal players like macrophages, adipose tissue, bioactive lipids, pro-inflammatory adipokines, inflammatory factors, and endoplasmic reticulum (ER) stress, shapes the delicate balance of physiological processes. This section delves into the symbiotic relationship between IR and inflammation, unraveling the molecular complexities and systemic implications.

### 5.1. Insulin Resistance Elicits an Inflammatory Response

De Sousa et al. first reported a potential association between inflammation biomarkers (serum haptoglobin and cortisol) and IR in cows [102]. They proposed that chronic inflammation may lead to IR, or that IR in feedlot cattle offered high-starch diets might be leading to chronic inflammation [102]. The observed potential association between inflammation biomarkers and IR holds particular significance in the context of dairy cows during the critical transition periods.

Tissue macrophages have been shown to produce leptin in human medicine [103], which in turn, leads to an increase in the concentrations of inflammatory markers (haptoglobin and cortisol) [104]. In dairy cows during early lactation, there is adipose-specific IR and high rates of lipid mobilization [12], and the infiltration of adipose tissue macrophages (ATM) is a response to this intense lipolysis [105,106]. Therefore, the role of macrophages represents a contributing factor to the promotion of inflammatory responses in IR in these transition cows. In the context of human IR, macrophages are recruited to insulin-sensitive tissues such as the liver and AT via chemokines (Figure 3) [107]. Their recruitment is mediated by the signaling of damage-associated molecular patterns (DAMP) affecting TLR on the macrophage surface, leading to the activation and translocation of nuclear factor κB (NF-κB) to the nucleus, where it acts as a transcription factor promoting the synthesis of pro-inflammatory factors including TNF-α, IL-1β, and IL-6 [108]. Concurrently, macrophages undergo polarization toward the M1 phenotype, leading to a pro-inflammatory state that further enhances the release of pro-inflammatory cytokines and chemokines (Figure 3) [109,110]. Under the influence of lipid accumulation, endothelial cells in AT increase the expression of adhesion proteins such as intercellular adhesion molecule-1 and vascular cell adhesion molecule-1, which in turn, promotes the migration of monocytes to the subendothelium and enhances their subsequent differentiation into macrophages [74,75]. This cascade of events intensifies the inflammatory environment within insulin-sensitive tissues, which contributes to the perpetuation of IR. However, while these events have not yet been fully validated and described in dairy cows, it has been found that during excessive lipolysis in transition dairy cows, intense ATM polarization to M1 takes place, finally accumulating in aggregates within omental and subcutaneous depots [111].

The greater impairment of insulin function is more likely to occur in dairy cows with more pronounced lipid mobilization [112], which inevitably accompanies the production of lipid factors and biologically active substances such as adiponectin, leptin, and Fibroblast growth factor-21 (FGF21). These products lead to varying degrees of impact on inflammatory responses.

Adiponectin, known as a potential anti-inflammatory marker, whose plasma concentration decreases during the first week postpartum in cows, can regulate the inflammatory response of cow macrophages by reducing TNF-α expression (Figure 3). Additionally, in human medicine, leptin exhibits pro-inflammatory characteristics and has been shown to activate recruited and resident immune cells, including macrophages, in AT, inducing the production of immune-related cytokines [113,114]. In dairy cows, leptin concentrations were high during late pregnancy and declined to a nadir at parturition [115]. Many studies have indicated that the greater the decline in body condition post-calving, the greater the reduction in plasma leptin concentration [115,116]. FGF21, a liver-synthesized factor, reaches peak plasma levels during calving in cattle, with white adipose tissue (WAT) being the primary target of FGF21 [117,118]. A recent study investigated the impact of the postpartum administration of recombinant FGF21 on early lactating cows [119]. In contrast to previous research reporting beneficial effects of exogenous FGF21 on insulin sensitivity in rodent models [120], FGF21 did not affect the plasma concentrations of insulin and adiponectin nor the response in the insulin concentration during a GTT in early lactating cows [119], but it did activate the ERK1/2 signaling pathway in white adipose tissue [121].

### 5.2. Inflammatory Mediators Contribute to the Development of Insulin Resistance

The multifactorial molecular mechanisms underlying inflammation-induced IR remain to be fully elucidated. During IR, mobilized immune cells, such as macrophages, release TNFα, IL-1β, and IL-6, which exert certain effects on metabolism. These factors can activate a series of intracellular signaling pathways, impair insulin signaling, and induce IR. In human medicine, it has been proven that pro-inflammatory cytokines activate the IKK/NF-κB pathway, JNK and other MAPKs, PKCs, and JAK/STAT/Suppressor of Cytokines Signaling Pathways [122,123]. These pathways are all involved in the regulation of insulin sensitivity and can contribute to IR when dysregulated, particularly in the context of obesity or high-fat diet feeding. Based on in vitro research using bovine adipocytes, it is currently known that TNF-α activates the NF-κB and JNK pathways, leading to phosphorylation of IRS-1 and IRS-2, thereby inhibiting insulin signal transduction [124].

Inflammatory mediators such as IL-1β, IL-6, and TNF-α are mutually regulated with endoplasmic reticulum (ER) stress [125]. In non-ruminant animals, ER stress is a significant contributor to impaired insulin signaling in the liver and adipose tissue [126,127]. Currently, it is known that ER stress is activated in tissues after parturition in cows [128]. In vitro experiments inducing ER stress in calf hepatocytes by β-hydroxybutyrate, revealed decreased phosphorylation of Akt and Glycogen Synthase Kinase 3β, as well as an upregulated abundance of gluconeogenic genes (phosphoenolpyruvate carboxykinase and glucose-6-phosphatase), indicating a state of IR. However, the addition of tauroursodeoxycholic acid in bovine hepatocytes to block ER stress may contribute to alleviating this condition [129]. However, further experimental validation is needed to determine whether reducing inflammatory mediators in hepatic cells directly affects the occurrence of ER and IR in the livers of dairy cows.

Additionally, resistin, as a pro-inflammatory adipokine [130,131], may serve as a mediator inducing IR in dairy cows (Figure 3). Resistin peaks in plasma during the first week postpartum in dairy cows [13,51]. In humans, resistin was suggested as a potential marker for IR [132], as studies have shown that elevated levels of resistin reduce the sensitivity of adipose tissue to insulin and promote the production of IL-6 and TNF-α [133]. In bovine AT explants, studies have demonstrated that resistin increases glycerol release and the mRNA levels of HSL and adipose triglyceride lipase (ATGL), indicating its potential to promote lipid mobilization [13]. However, more extensive investigations in dairy cows are warranted to elucidate its effects on the insulin signaling pathway.

## 6. Conclusions

Insulin resistance is a complex metabolic disorder observed in transition dairy cows as they prepare for the shift from pregnancy to lactation. During this critical period, IR plays a pivotal role in facilitating the flow of glucose to essential organs such as the pregnant uterus, mammary gland, and immune cells, supporting their increased metabolic demands. This adaptation is vital for maintaining blood glucose homeostasis and ensuring optimal energy distribution even during times of peak lactation. Insulin resistance in transition cows is influenced by factors like hormonal fluctuations, heightened lipolysis, and altered glucose metabolism, all of which contribute to the risk of metabolic disorders such as ketosis. Metabolic inflammation in transition dairy cows results from hormonal changes, increased lipolysis, and altered glucose metabolism. This leads to elevated NEFA release, the activation of inflammatory cells, and the upregulation of pro-inflammatory cytokines. In over-conditioned cows, adipose tissue inflammation exacerbates inflammatory pathways, impairing insulin signaling and worsening metabolic inflammation. Moreover, IR is intricately linked to metabolic inflammation, wherein enhanced lipolysis, immune activation, and insulin signaling pathways interact to create a pro-inflammatory environment. Understanding the multifactorial nature of IR and its interplay with metabolic inflammation is crucial for managing the health, welfare, and productivity of transition dairy cows. To prevent the occurrence of NEB, the following may provide direction for prospective research areas in the future: (1) Nutrition: Research should be conducted on novel feed ingredients and dietary techniques to support milk production, while minimizing energy deficits and negative energy balance. (2) Health Monitoring: Advanced monitoring technologies, including wearable sensors and precision diagnostics, should be utilized for the early detection and management of metabolic disorders such as ketosis, fatty liver, and displaced abomasum, since they are crucial for preventing and mitigating negative energy balances. (3) Breeding: Continued investigations should be conducted into the inheritance of energy balance traits to develop genomic selection tools for breeding dairy cows with improved energy metabolism and reduced susceptibility to negative energy balance. (4) Reproduction: Researchers should continue the refinement and development of precision reproductive technologies, synchronization methods, and the optimized use of reproductive hormones to enhance conception rates and shorten calving intervals.

## Data Availability

Not applicable.
